# Machine Learning Algorithm to Predict Worsening of Flexion Range of Motion After Total Knee Arthroplasty

**DOI:** 10.1016/j.artd.2022.07.011

**Published:** 2022-08-19

**Authors:** Yoshitomo Saiki, Tamon Kabata, Tomohiro Ojima, Shogo Okada, Seigaku Hayashi, Hiroyuki Tsuchiya

**Affiliations:** a Department of Orthopaedic Surgery, Graduate School of Medical Sciences, Kanazawa University, Ishikawa, Japan; b Department of Rehabilitation Physical Therapy, Faculty of Health Science, Fukui Health Science University, Fukui, Japan; c Department of Orthopaedic Surgery, Fukui General Hospital, Fukui, Japan; d Division of Advanced Science and Technology, Japan Advanced Institute of Science and Technology, Ishikawa, Japan

**Keywords:** Range of motion, Total knee arthroplasty, Machine learning, Random forest

## Abstract

**Background:**

Predicting the worsening of flexion range of motion (ROM) during the course post-total knee arthroplasty (TKA) is clinically meaningful. This study aimed to create a model that could predict the worsening of knee flexion ROM during the TKA course using a machine learning algorithm and to examine its accuracy and predictive variables.

**Methods:**

Altogether, 344 patients (508 knees) who underwent TKA were enrolled. Knee flexion ROM worsening was defined as ROM decrease of ≥10° between 1 month and 6 months post-TKA. A predictive model for worsening was investigated using 31 variables obtained retrospectively. 5 data sets were created using stratified 5-fold cross-validation. Total data (n = 508) were randomly divided into training (n = 407) and test (n = 101) data. On each data set, 5 machine learning algorithms (logistic regression, support vector machine, multilayer perceptron, decision tree, and random forest) were applied; the optimal algorithm was decided. Then, variables extracted using recursive feature elimination were combined; by combination, random forest models were created and compared. The accuracy rate and area under the curve were calculated. Finally, the importance of variables was calculated for the most accurate model.

**Results:**

The knees were classified into the worsening (n = 124) and nonworsening (n = 384) groups. The random forest model with 3 variables had the highest accuracy rate, 0.86 (area under the curve, 0.72). These variables (importance) were joint-line change (1.000), postoperative femoral-tibial angle (0.887), and hemoglobin A1c (0.468).

**Conclusions:**

The random forest model with the above variables is useful for predicting the worsening of knee flexion ROM during the course post-TKA.

## Introduction

Total knee arthroplasty (TKA) is an effective surgical procedure for pain relief and functional restoration in patients with advanced knee osteoarthritis and rheumatoid arthritis. After TKA, it is important to achieve and maintain a functional range of motion (ROM). Poor flexion ROM reduces patient’s activities of daily living ability and leaves the patient’s preoperative expectations unmet post-TKA [[1], [2], [3]]. Therefore, good ROM is an important goal to achieve and maintain.

Postoperative knee flexion ROM is affected by preoperative, intraoperative, and postoperative factors such as age, sex, obesity, diabetes, preoperative ROM, and joint-line changes [[4], [5], [6], [7], [8], [9]]. There are cases where the flexion ROM, once acquired in the early postoperative period, gradually worsens during the course post-TKA [9,10]. Predicting this worsening of flexion ROM may play a vital role in postoperative follow-up and rehabilitation and help improve patients’ activities of daily living ability and meet their expectations. Therefore, predicting the worsening of flexion ROM during the course post-TKA is clinically meaningful.

Recently, the number of reports using machine learning (ML) for accurate predictions has increased in the field of total joint arthroplasty [[11], [12], [13], [14], [15]]. ML is a theoretical system used to create models for predicting unknown data from past data. ML allows us to select an optimal algorithm from various predictive methods and to create the best predictive model. Moreover, ML algorithms can extract variables (input features) that contribute to the prediction of the target variable (herein, the worsening of flexion ROM). ML differs from conventional statistical methods in that the algorithms are designed to improve the predictive accuracy of future data from past data. Therefore, compared with conventional methods, it can be expected to construct a predictive model that is more robust with respect to unknown data.

This retrospective study aimed to investigate the algorithm with the highest predictive performance for the worsening of knee flexion ROM during the course post-TKA, to create a predictive model with the highest predictive performance, to verify its predictive accuracy, and to investigate which variables are necessary for the accurate prediction.

## Material and methods

### Ethical information

This retrospective study was approved by the institutional review board and conducted according to the principles of the Declaration of Helsinki. Informed consent was obtained from all patients for the use of their information.

### Study design and patients

This retrospective study included 401 consecutive patients (578 knees), in whom primary TKA was performed at our center, from April 2017 to July 2021. We excluded 23 patients (30 knees) who could not be followed up until 6 months post-TKA from the analyses.

### Procedure and rehabilitation protocols

All TKAs were performed by the same single surgeon. The surgery was performed using a standard medial parapatellar approach or lateral parapatellar approach. Six patients (7 knees) who had a lateral parapatellar approach were excluded. Different prostheses were utilized: Logic knee system (234 knees; Exactech Inc., Gainesville, FL), Persona knee system (221 knees; Zimmer Biomet Inc., Warsaw, IN), or FINE knee system (53 knees; Nakashima Medical Inc., Okayama, Japan). The FINE knee system was used with an antimicrobial coating for patients at relatively high risk of infection. Different implant surfaces were used, namely cruciate-retaining, posterior-stabilizing, cruciate-substituting, bicruciate-retaining, or medial-congruent implants. Twenty-eight patients (33 knees) with a non–cruciate-retaining design were excluded. All patients underwent the same postoperative rehabilitation program, which was started with standard postoperative ROM exercises, as tolerated, at 1 day postoperatively.

### Radiological measurements

The anteroposterior and lateral radiographs of the knees were obtained preoperatively and immediately after surgery in all patients. Four component angles (α, β, γ, and θ) were measured on the postoperative radiographs [16,17]. The α-angle was defined as the internal angle between the parallel line with the femoral condyles and the femoral shaft axis in the anteroposterior radiographs. The β-angle was defined as the internal angle between the parallel line to the plateau of the metal tibial component and the tibial shaft axis in the anteroposterior radiographs. The γ-angle was defined as the angle between the line perpendicular line to the distal metal-cement interface of the femoral component and the femoral shaft axis in the lateral radiographs. The θ-angle was defined as the angle between the parallel line to the plateau of the metal tibial component and the tibial shaft axis in the lateral radiographs. We measured the height of the joint line using the method described by Figgie et al. [18], which is the distance from the top of the tibial tuberosity to the tibial plateau on the preoperative lateral radiographs or from the top of the tibial tuberosity to the most distal femoral component on the postoperative lateral radiographs. The joint-line change was calculated as the difference between the preopertaive and postoperative height of the joint line. Moreover, at discharge, coronal long-leg radiographs were taken, for which the patient stood with the limb in neutral rotation, the patella facing forward, and the knee extended. The femoral-tibial angle (FTA) was measured on long-leg radiographs. In all radiological measurements, the intraclass correlation coefficients for intrarater reliability were in the range of 0.93-0.99, and the intraclass correlation coefficients for interrater reliability were in the range of 0.72-0.92.

### Primary outcome and predictive variables

The primary outcome was the changes in knee flexion ROM during the course post-TKA. Since, according to a previous study, evident improvement in ROM continues during the first 6 months post-TKA [19], we checked for the worsening of flexion ROM at this time. The worsening of flexion ROM was defined as a decrease of ≥10° in the flexion ROM from 1 month to 6 months post-TKA; it was recorded as a binary variable (worsening or nonworsening). A study reported that the minimum significant difference in knee ROM measurement using a goniometer is ≥ 10° [20]. Therefore, in this study, the cutoff value for the worsening of flexion ROM was set at 10°. The knee flexion ROM was passively measured using a long goniometer at 1 month and 6 months post-TKA. The patients assumed the supine position, and the flexion ROM was measured with reference to bone landmarks, including the greater trochanter, lateral epicondyle of the femur, and lateral malleolus. Selection bias would have occurred if we excluded patients who, within 6 months post-TKA, underwent surgical manipulation under anesthesia. Therefore, for such patients, flexion ROM data were recorded immediately before manipulation under anesthesia as data at 6 months post-TKA.

We retrospectively collected 31 variables, which were divided into the following 3 categories: (1) preoperative, (2) intraoperative, and (3) postoperative variables. The preoperative variables included patient’s age, sex, body mass index, operated side, primary disease (osteoarthritis or rheumatoid arthritis), history of ipsilateral knee procedure, hemoglobin A1c (HbA1c), FTA, active/passive knee flexion ROM, active/passive knee extension ROM, comfortable/maximum gait speed, and Japanese Orthopedic Association score. The Japanese Orthopedic Association score, an orthopedic method for assessing physical function, consists of 100 points, with higher scores indicating better function [[21], [22], [23]].

The intraoperative variables (obtained during or immediately post-TKA) included prosthetic component (Logic, Persona, or FINE), α-angle, β-angle, γ-angle, θ-angle, and joint-line changes.

The postoperative variables were the FTA at discharge, active/passive knee flexion ROM, active/passive knee extension ROM, comfortable/maximum gait speed, and Japanese Orthopedic Association score at 1 month post-TKA.

### Statistical analyses

**Figure 1:**
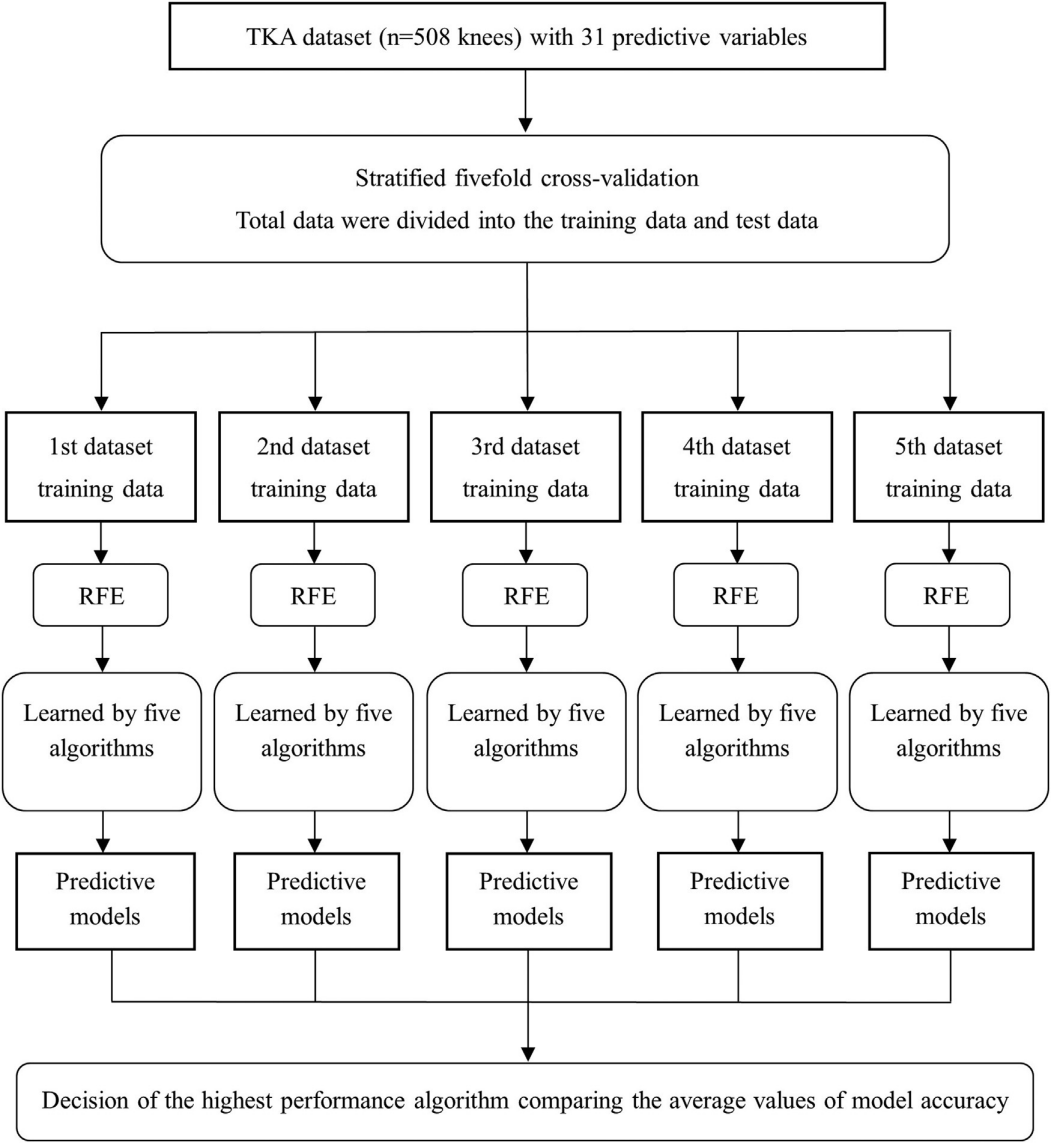
Flow of the first analysis.

**Figure 2:**
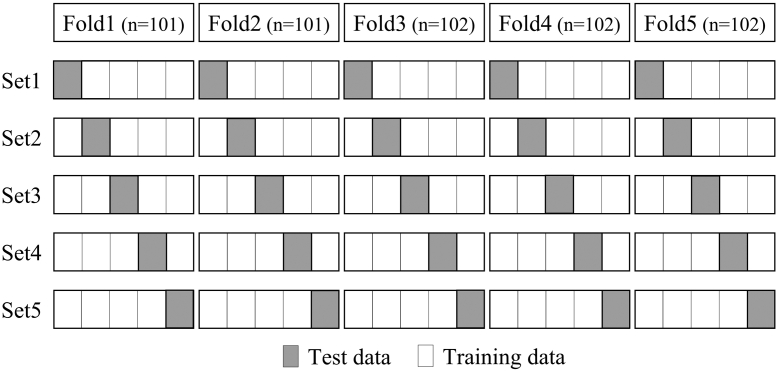
Summary chart of stratified 5-fold cross-validation.

**Figure 3:**
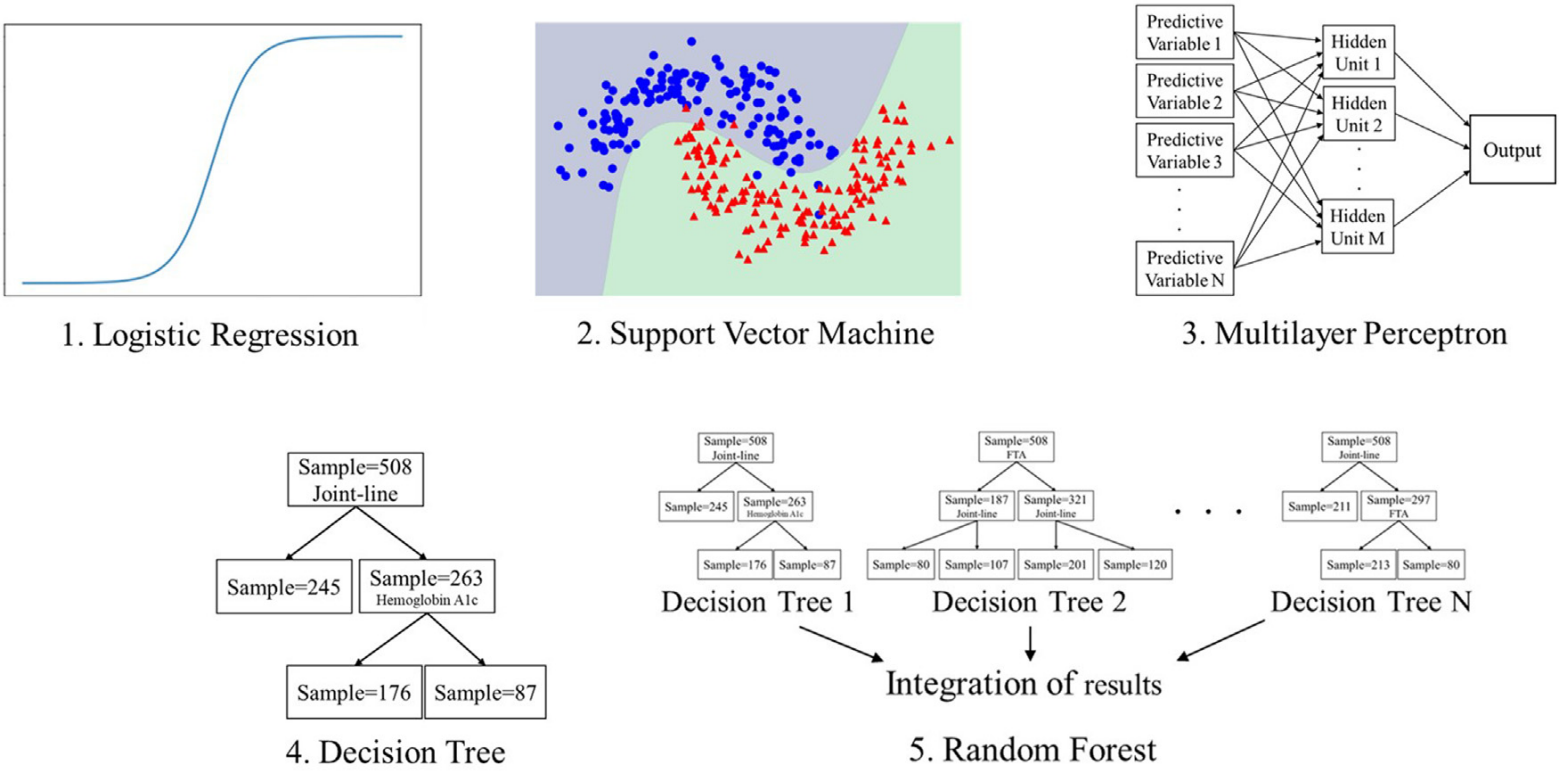
5 machine learning algorithms.

**Figure 4:**
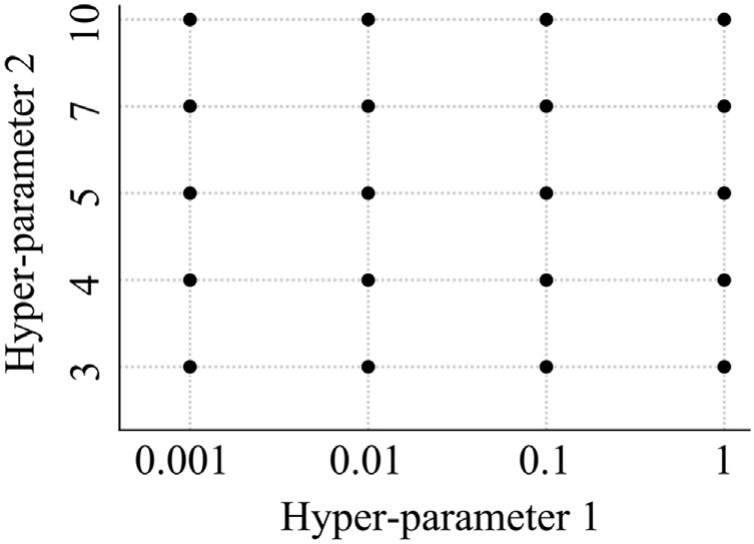
Summary chart of hyperparameter tuning using the grid search.

In total, 94 of 16,256 records (0.6%) were incomplete. Incomplete data were fully imputed using multiple imputation by chained equation [24]. First, we investigated an algorithm that could accurately predict the worsening of knee flexion ROM. The procedure for this investigation is outlined in Figure 1. Cross-validation enables us to validate how the model will perform on an unknown data set (test data set, ie, not used for training) and, so, it is used for a more accurate evaluation of model prediction performance. We created 5 data sets using stratified 5-fold cross-validation (Fig. 2). In the stratified 5-fold cross-validation, all data were divided equally and randomly into 5 folds, and each fold was further divided into 5 groups. One test data was selected for each fold, and the remaining 4 were used as training data. The test and training data within the 5 folds were added together to create 1 data set. Each group was used once as the test data and 4 times as the training data to create 5 data sets. For each data set, 5 variables, considered useful for predicting the worsening of flexion ROM, were extracted from 31 variables using recursive feature elimination with the random forest algorithm. Then, we attempted to create a model that could predict the worsening of flexion ROM. We applied the following 5 ML algorithms to each data set: (1) logistic regression, (2) support vector machine, (3) multilayer perceptron, (4) decision tree, and (5) random forest (Fig. 3). Logistic Regression is an algorithm for classifying data by linear separation. Support vector machine is an algorithm for optimal classification based on the distance between the boundary surface and the respective data. Multilayer Perceptron is an algorithm for creating complex and flexible prediction models by using hidden layers and nonlinear activation functions. Decision tree is an algorithm for classifying data by learning simple decision rules inferred from the data features. Random forest is an algorithm for using bootstrap aggregating to create multiple decision trees. Next, we performed hyperparameter tuning using the grid search method and created predictive models for each dataset. Grid search is an exhaustive algorithm to find the best combination of hyperparameters (Fig. 4). We divided the domain of the hyperparameters into a grid. We tried every combination of values of this grid and compared the values of the area under the curve (AUC) calculated in these combinations. The point of the grid with the maximum AUC was assumed the best combination of hyperparameters. Finally, to assess the performance of predictive models, the average accuracy rate and AUC were calculated for each algorithm. The AUCs of 0.6-0.7, 0.71-0.8, and >0.8 were interpreted as weak, satisfactory, and strong predictive performance, respectively [25]. The aforementioned method was effective for creating a predictive model that was robust to outliers and reduced the risk of overfitting.

Second, we investigated the optimal combination of variables to create the best predictive model. For the same 5 data sets used earlier, we set 4 conditions for variable extraction by recursive feature elimination, such as conditions with the superior 5, 4, 3, or 2 variables. For each condition, predictive models were created using the random forest, which had exhibited the highest predictive accuracy in the first investigation. We performed hyperparameter tuning using the grid search method. The average accuracy rate and AUC for each condition were calculated to assess the performance of predictive models.

Third, we calculated the importance of variables based on the results of the first and second investigations. The importance of each variable indicated the degree of influence in predicting the worsening of flexion ROM. From the same 5 data sets used earlier, 3 superior variables, that is, the condition of variables with the highest predictive accuracy in the second investigation, were extracted using recursive feature elimination. Then, the importance of these variables for 5 data sets was calculated using a random forest algorithm. The average importance of variables was calculated for each variable. It was standardized to a minimum value of 0 and a maximum value of 1 and then averaged for all data sets. The codes to perform analysis were written using Python [26]. We have publicly released the code for reproducibility (https://github.com/yoshitomosaiki/flexion_change_prediction).

## Results

### Summary of predictive variables for the worsening and nonworsening groups

In total, 344 patients (508 knees) were analyzed. Table 1 shows a summary of the 31 predictive variables for the worsening (n = 124) and nonworsening (n = 384) groups. The knee flexion ROM at 6 months post-TKA was 111.3 ± 12.2° in the worsening group and 123.2 ± 9.2° in the non-worsening group (*P* < .001).

**Table 1:** Summary of 31 predictive variables (worsening vs nonworsening).

Variables	Worsening	Nonworsening	*P* value
	n = 124	n = 384	
Preoperative variables			
Age (y)	74.1 (7.8)	74.1 (6.6)	.980
Sex (female, %)	71.0, 88	79.7, 306	.048
Body mass index (kg/m^2^)	25.8 (3.7)	25.6 (4.2)	.696
Operated side (right, %)	53.2, 66	51.0, 196	.681
Primary disease (osteoarthritis, %)	93.5, 116	91.1, 350	.458
Prior ipsilateral knee procedure (%)	4.0, 5	2.1, 8	.322
Hemoglobin A1c (%)	6.1 (0.7)	5.9 (0.5)	.001
Femoral-tibial angle (°)	185.5 (5.7)	184.6 (7.2)	.199
Active flexion ROM (°)	119.0 (17.9)	121.3 (16.1)	.180
Passive flexion ROM (°)	124.2 (17.7)	126.5 (16.0)	.177
Active extension ROM (°)	−10.2 (8.5)	−9.7 (7.4)	.530
Passive extension ROM (°)	−8.4 (8.1)	−8.3 (7.3)	.891
Comfortable gait speed (m/sec)	0.85 (0.28)	0.86 (0.30)	.547
Maximum gait speed (m/sec)	1.07 (0.39)	1.07 (0.38)	.977
JOA score (points)	57.6 (13.6)	60.1 (11.6)	.047
Intraoperative variables			
Prosthetic component (Logic, %)	42.7, 53	47.1, 181	.409
Prosthetic component (Persona, %)	49.2, 61	41.7, 160	.146
Prosthetic component (FINE, %)	8.1, 10	11.2, 43	.399
α-angle (°)	96.8 (2.4)	96.5 (5.0)	.554
β-angle (°)	89.7 (1.2)	89.3 (4.4)	.265
γ-angle (°)	3.7 (2.2)	3.8 (2.0)	.702
θ-angle (°)	86.5 (2.0)	86.8 (1.8)	.136
Joint-line change (mm)	3.0 (3.1)	1.5 (2.2)	<.001
Postoperative variables			
Femoral-tibial angle (°)	174.0 (2.1)	175.1 (1.8)	<.001
Active flexion ROM (°)	117.6 (10.7)	117.3 (10.0)	.763
Passive flexion ROM (°)	124.2 (10.4)	123.1 (9.5)	.177
Active extension ROM (°)	−7.1 (6.4)	−5.7 (5.1)	.010
Passive extension ROM (°)	−2.5 (3.3)	−1.9 (3.2)	.107
Comfortable gait speed (m/sec)	0.81 (0.22)	0.82 (0.23)	.851
Maximum gait speed (m/sec)	1.00 (0.28)	0.99 (0.30)	.532
JOA score (points)	71.3 (10.2)	73.4 (10.0)	.042

JOA, Japanese Orthopedic Association.

Values are presented as mean (standard deviation) or percentage, number.

### Algorithm selection based on the predictive accuracy

Table 2 shows the average accuracy rate and AUC for the 5 ML models. Of the 5 models, the random forest had the highest accuracy rate (0.84) and AUC (0.71).

**Table 2:** Algorithm selection based on the predictive accuracy.

Algorithms	Accuracy rate	AUC
Logistic regression	0.81 (0.77-0.86)	0.63 (0.49-0.76)
Support vector machine	0.81 (0.76-0.86)	0.63 (0.50-0.77)
Multilayer perceptron	0.80 (0.77-0.83)	0.61 (0.50-0.72)
Decision tree	0.82 (0.78-0.86)	0.69 (0.56-0.81)
Random forest	0.84 (0.81-0.88)	0.71 (0.58-0.83)

Values are presented as mean (95% confidence interval).

### Optimal predictive variables and their importance

**Figure 5:**
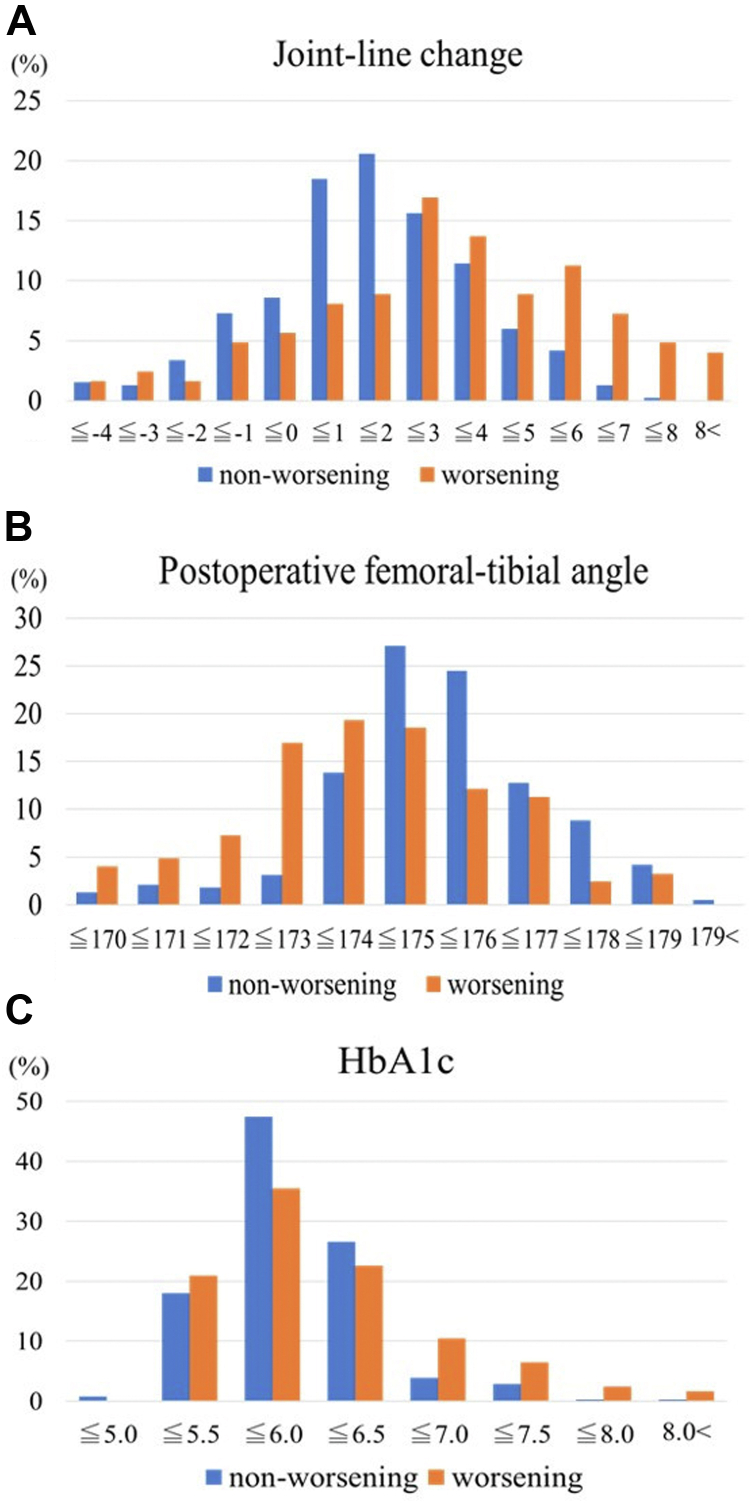
Distribution of the variables. (a) Distribution of joint-line change for the 2 groups. (b) Distribution of postoperative femoral-tibial angle for the 2 groups. (c) Distribution of HbA1c for the 2 groups.

Table 3 shows the average accuracy rate and AUC for 5 combinations of variables, such as the superior 5, 4, 3, or 2 variables. The random forest model with 3 variables had the highest accuracy rate (0.86) and AUC (0.72). Table 4 shows the importance of variables in the best predictive model. The importance of the 3 variables was as follows: joint-line change (1.000), postoperative FTA (0.887), and HbA1c (0.468). Figure 5 shows the distribution of these variables.

**Table 3:** Optimal predictive variables.

Combinations of variables	Accuracy rate	AUC
5 variables	0.84 (0.81-0.88)	0.71 (0.58-0.83)
Four variables	0.85 (0.81-0.88)	0.72 (0.59-0.84)
Three variables	0.86 (0.81-0.89)	0.72 (0.59-0.85)
Two variables	0.84 (0.80-0.87)	0.70 (0.58-0.82)

Values are presented as mean (95% confidence interval).

**Table 4:** Importance of variables in the best predictive model.

Variables	Importance
Joint-line change	1.000
Femoral-tibial angle (postoperative)	0.887
Hemoglobin A1c	0.468

## Discussion

This study demonstrates that the random forest model with 3 variables, including joint-line change, postoperative FTA, and HbA1c, had a satisfactory predictive performance and was suitable for predicting the worsening of knee flexion ROM during the course post-TKA.

The main difference between ML and traditional statistical methods is its purpose. ML models estimate model parameters from data sets and are created to improve the predictive accuracy of future data as much as possible, whereas statistical models determine associations between variables in a data set. Therefore, in multiple regression analysis, weights that are overfitted to the data set are learned when trying to predict future data using a regression function that has overfitting weight parameters, and the estimation accuracy is often reduced. The random forest algorithm can create highly representative predictive models by performing nonlinear separation, and use bootstrap aggregating to create multiple decision trees, which are weak classifiers constructed by a combination of different features [27]. At the time of prediction, the output results of multiple weak classifiers with different properties are integrated. Moreover, in decision trees and random forests, decision rules suitable for classification are selected based on the results of statistical analysis, and features that are not suitable for classification are removed internally. The other algorithms do not have an effective feature selection function, which may have reduced their accuracy. We consider that these representativeness, bootstrap aggregating and feature selection were the reason for the higher prediction accuracy in the random forest algorithm than in the other 4 algorithms. Therefore, high predictive performance was obtained because a solution with high certainty can be determined. Additionally, in ML, it is important to verify properly the performance of the predictive model for unknown data. Therefore, stratified k-fold cross-validation was used. In this method, different combinations of training data and test data are created, and a model is created and averaged for each combination; thus, the performance of the predictive model can be appropriately verified. Hence, it is considered that the construction of the predictive model of this study and its accuracy verification were performed appropriately.

In the ML model, variables are selected to maximize the prediction accuracy. The problem is that it is difficult to interpret the relationship between the target and predictor variables and the reasons for the prediction. However, to increase the transparency of the predictive model, it is necessary to review the data and discuss the reasons for the predictions. In this study, the predictive variables in the best predictive model were joint-line change, postoperative FTA, and HbA1c. Previous studies have reported that excessive joint-line elevation of more than 4-5 mm may decrease flexion ROM [18,28,29]. The present study also showed a significant increase in the joint line in the worsening group. Furthermore, Figure 2a shows a low worsening probability with an increase of 0-2 mm and a high worsening probability with an increase of >4 mm. Since patients with a higher joint line require more stretching of the knee joint extensor muscles during flexion, we believe that the ROM once acquired through intensive rehabilitation during hospitalization will decrease if the same level of stretching load is not given after discharge. There are few reports on postoperative FTA and flexion angle. Figure 2b confirms a high worsening probability for patients with valgus >173°. Joint laxity may have an effect, but it is difficult to clarify the relationship between postoperative FTA and worsening of the flexion angle from this study. Previous studies have shown that patients with diabetes mellitus are likely to decrease the knee flexion ROM post-TKA [8,9]. Figure 2c shows that the worsening probability of the flexion angle is particularly high in patients with HbA1c 5.5-6.0 or >6.5, which suggests that HbA1c contributes to the prediction of cases with relatively high values. Therefore, we believe that joint-line change, postoperative FTA, and HbA1c are valid predictors. Moreover, these variables have high availability in that they are often obtained in daily clinical practice. However, some variables still require investigation, such as component rotational alignment and psychological factors. Component malrotation reportedly causes arthrofibrosis and stiffness [30,31]. Furthermore, we could not evaluate psychological factors. Psychological factors, such as kinesiophobia and neglect-like symptoms, were reported to affect flexion ROM post-TKA [32,33]. The use of these variables may improve the predictive performance in the future.

The 3 variables extracted in this study can be obtained early post-TKA in daily practice. Therefore, the policy of rehabilitation after discharge can be determined according to the predicted results. Fleischman et al. [34] reported that home exercise rehabilitation was noninferior to professional supervised rehabilitation in flexion ROM at 6 months post-TKA, while recommending supervised rehabilitation for patients with poor recovery. Our predictive model may act as a reference for postdischarge rehabilitation strategy. If predicted as the nonworsening type, home exercise rehabilitation could be initiated subsequent to hospital discharge, whereas if predicted as the worsening type postdischarge, supervised rehabilitation could be provided for 3-6 months post-TKA [19].

One of the strengths of this study is that it may aid in developing a method for predicting postoperative outcomes using ML. Recently, ML has been introduced in the medical field, and its further expansion and development are expected. Second, the results of this study suggest that the worsening of flexion ROM during the course post-TKA can be predicted with fewer variables at an early postoperative stage. Third, although this was a retrospective study, the number of missing data was small, and bias was reduced as much as possible using multiple imputations.

The study also has a few limitations. First, the predictive performance may be improved as discussed previously. Unexamined variables may improve predictive performance and should be further investigated. Second, the study could not be externally validated. Although we have decreased the analysis error using stratified 5-fold cross-validation, data were obtained from a single center. In the future, based on the attached code, the performance of our predictive model should be verified at multiple centers.

## Conclusions

We created a model that predicted the worsening of knee flexion ROM during the course post-TKA using an ML algorithm and examined its accuracy and predictive variables. We found that the random forest algorithm with 3 variables, including joint-line change, postoperative FTA, and HbA1c produced the prediction model with satisfactory accuracy for flexion ROM deterioration from 1 to 6 months after TKA. This predictive model may be highly useful and available for postdischarge rehabilitation strategies after TKA because on these 3 variables can be obtained early post-TKA in daily practice.

## Conflicts of interest

The authors declare there are no conflicts of interest.

For full disclosure statements refer to https://doi.org/10.1016/j.artd.2022.07.011.

## Author contributions

All authors meet the following 4 criteria. Substantial contributions to the conception or design, acquisition, analysis, or interpretation of data for the work. Drafting the work or revising it critically for important content. Final approval of the version to be published. Agreement to be accountable for all aspects of the work in ensuring that questions related to the accuracy or integrity of any part of the work are appropriately investigated and resolved.

## Appendix A Supplementary data

Conflict of Interest Statement for Hayashi

Conflict of Interest Statement for Kabata

Conflict of Interest Statement for Ojima

Conflict of Interest Statement for Okada

Conflict of Interest Statement for Saiki

Conflict of Interest Statement for Tsuchiya
